# Editorial: Advancements and optimization of evidence-based approaches in pain management

**DOI:** 10.3389/fpubh.2026.1896780

**Published:** 2026-07-21

**Authors:** Areerat Suputtitada, J. Douglas Thornton, Marcos Brioschi

**Affiliations:** 1 Department of Rehabilitation Medicine, Faculty of Medicine, Chulalongkorn University, Bangkok, Thailand; 2 Principles and Practice of Clinical Research (PPCR) Program, Harvard T.H. Chan School of Public Health, Harvard University, Boston, MA, United States; 3 Department of Pharmaceutical Health Outcomes and Policy, University of Houston College of Pharmacy, Houston, TX, United States; 4 Neurology Department, University of São Paulo, São Paulo, Brazil

**Keywords:** biopsychosocial model, function-oriented care, neuroscience-informed care, non-pharmacological interventions, pain management, precision rehabilitation, public health, rehabilitation medicine

Pain management remains one of the most important and challenging fields in modern healthcare. Chronic musculoskeletal pain, spinal disorders, perioperative pain, neuropathic syndromes, and other persistent pain conditions continue to impose substantial individual, societal, and economic burdens worldwide. Despite growing evidence supporting biopsychosocial and interdisciplinary approaches, pain management in many healthcare systems remains heavily centered on pharmacological, procedural, and structurally focused interventions, often with insufficient integration of rehabilitation, psychological support, physical activity, and long-term functional restoration.

This Research Topic was originally developed in response to the urgent need for a broader and more evidence-based paradigm in pain care. The goal was not only to explore advancements in pain management, but also to challenge prevailing narratives that frequently prioritize symptom suppression over restoration of movement, function, participation, and quality of life. The Research Topic further sought to promote discussion regarding balanced integration among medical, non-pharmacological, rehabilitative, behavioral, and systems-based approaches while emphasizing cost-effectiveness, sustainability, and real-world clinical implementation.

At the same time, pain science itself has undergone a major conceptual transformation. Pain is increasingly recognized not simply as a peripheral nociceptive phenomenon, but as a multidimensional biopsychosocial and neurophysiological experience shaped by interactions among tissue pathology, nervous system plasticity, psychological state, behavior, environment, and social context. This evolving understanding has accelerated the transition from isolated symptom-centered models toward evidence-based, neuroscience-informed, interdisciplinary, and function-oriented care.

The articles included in this Research Topic collectively reflect this important shift in contemporary pain medicine.

Several manuscripts emphasized the expanding role of non-pharmacological and rehabilitation-oriented interventions in pain management. Acupuncture-based therapies (Zhang Y. et al.; Hong et al.; Allam et al.), laser acupuncture (Hong et al.; Allam et al.), moxibustion (Wang et al.), Fu's subcutaneous needling (Shao et al.), psychosensory interventions (Bustamante Fernández et al.), and multimodal rehabilitation strategies (Chen and Suputtitada; Cross et al.) demonstrated promising effects on pain reduction, functional recovery, emotional wellbeing, and patient-centered outcomes. These findings reinforce the increasing importance of integrating evidence-based non-pharmacological approaches into comprehensive pain care pathways.

Another major theme involved neuroscience-informed and biopsychosocial approaches to pain modulation. Multiple studies highlighted the importance of psychological, sensory, emotional, and neurophysiological mechanisms in shaping pain perception and recovery. Psychosensory interventions for pediatric procedural pain (Bustamante Fernández et al.), behavioral strategies, and patient-centered rehabilitation approaches (Chen and Suputtitada; Cross et al.) collectively reflect the growing recognition that chronic pain extends beyond tissue pathology alone and involves complex interactions among central sensitization, neuroplasticity, cognition, emotion, and behavior.

Perioperative and acute pain optimization also emerged as an important focus within this Research Topic. Studies evaluating opioid-sparing strategies (Ouyang et al.; Meng et al.), patient-controlled analgesia optimization (Chen et al.; Meng et al.), healthcare failure mode and effect analysis (Chen et al.), and multimodal perioperative pain management (Chen et al.; Ouyang et al.; Meng et al.) demonstrated the increasing emphasis on improving postoperative recovery, reducing complications, enhancing patient safety, and improving patient-reported outcomes.

Systems-based and digitally enabled models of care additionally represented an important direction within this Research Topic. Virtual musculoskeletal integrated practice units (Cross et al.), integrated rehabilitation pathways (Chen and Suputtitada), and technology-assisted healthcare delivery models (Cross et al.) highlighted the growing importance of scalable, accessible, and coordinated systems capable of addressing persistent barriers in pain management, including fragmented care, limited specialist access, geographic disparities, and rising healthcare costs.

Public health and epidemiological perspectives further reinforced the substantial global burden of low back pain and chronic musculoskeletal disorders (Zheng et al.; Zhang C. et al.). These findings underscore the urgent need for sustainable, function-oriented, and population-level approaches to pain prevention and management while supporting stronger integration of rehabilitation medicine into healthy aging and public health strategies.

Emerging regenerative and mechanobiological approaches additionally represent an evolving area of interest in pain and rehabilitation medicine. Recent translational and clinical evidence, including mechanobiological perspectives on ultrasound-guided mechanical needling with sterile water injection (Chen and Suputtitada), suggests potential roles in fibrosis modulation, calcification reduction, tissue mobility restoration, neuromodulation, and functional recovery. Further real-world, longitudinal, and implementation-focused investigations may help optimize patient selection, procedural standardization, long-term outcome evaluation, and integration into evidence-based rehabilitation and pain management frameworks.

Such approaches reflect the broader movement toward minimally invasive, biologically informed, and function-oriented interventions within contemporary pain and rehabilitation medicine. This evolving paradigm is illustrated in Figure 1, which highlights the transition from fragmented, pharmacology-focused approaches toward interdisciplinary, multimodal, and patient-centered strategies emphasizing function, participation, independence, and quality of life. Advances in pain neuroscience, rehabilitation science, psychosocial medicine, regenerative rehabilitation, implementation science, and public health are collectively reshaping pain care toward more personalized, evidence-based, and integrated frameworks. Evidence-based interventions highlighted in this Research Topic include acupuncture-based therapies (Zhang Y. et al.; Hong et al.; Allam et al.), psychosensory and behavioral approaches (Bustamante Fernández et al.), perioperative optimization (Chen et al.; Ouyang et al.; Meng et al.), digital rehabilitation models (Cross et al.), and emerging regenerative interventions, including ultrasound-guided mechanical needling with sterile water injection for fibrosis and calcification reduction, tissue mobility restoration, and functional recovery (Chen and Suputtitada).

**Figure 1:**
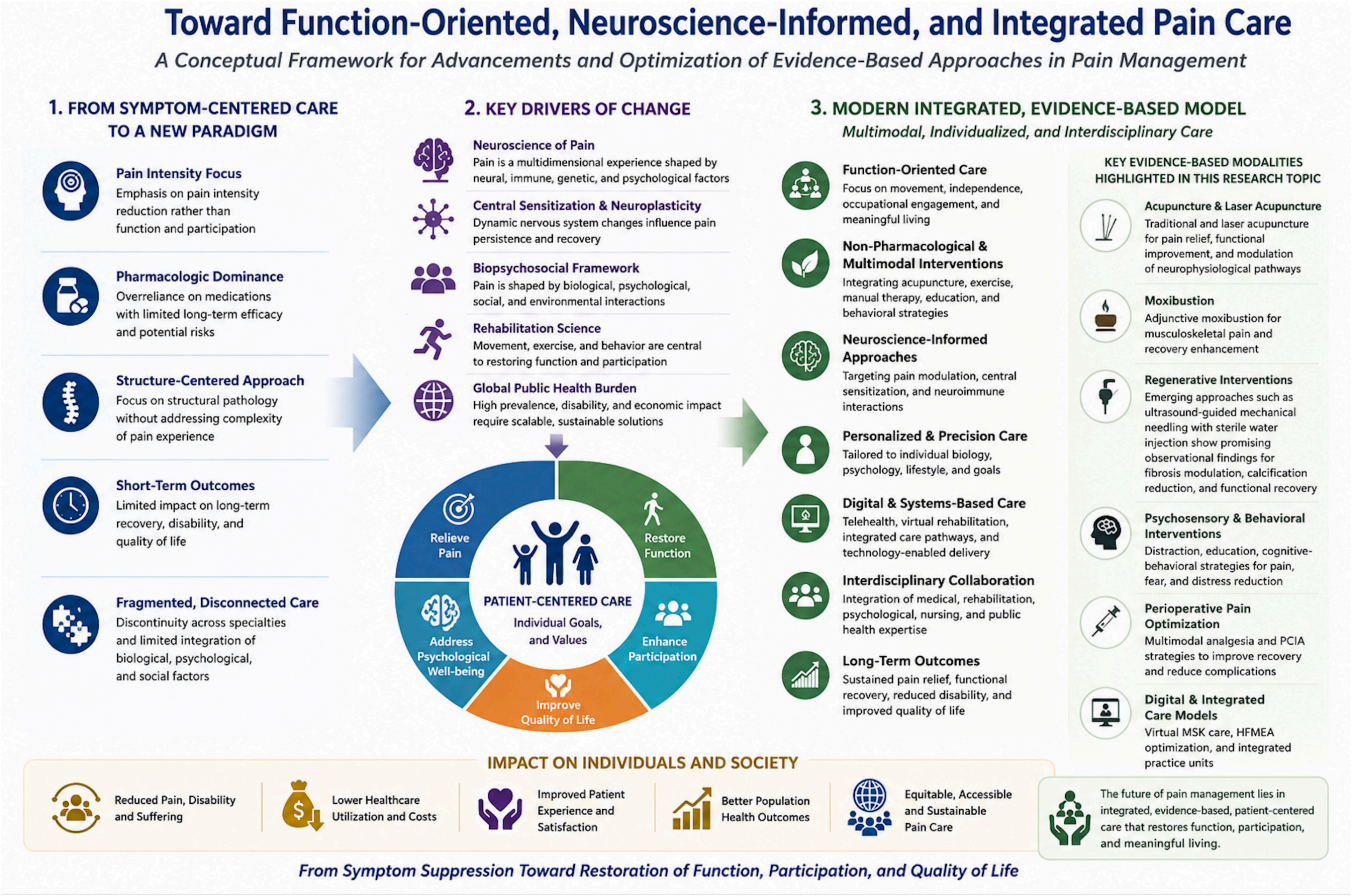
Transition from symptom-centered pain management toward function-oriented, neuroscience-informed, and integrated pain care. The figure illustrates the evolving shift from fragmented, pharmacology-focused approaches toward interdisciplinary, multimodal, and patient-centered strategies emphasizing functional restoration, participation, independence, and quality of life. Evidence-based interventions highlighted in this Research Topic include acupuncture-based therapies (Zhang Y. et al.; Hong et al.; Allam et al.), psychosensory and behavioral approaches (Bustamante Fernández et al.), perioperative optimization (Chen et al.; Ouyang et al.; Meng et al.), digital rehabilitation models (Cross et al.), and emerging procedural strategies including ultrasound-guided mechanical needling with sterile water injection that support tissue recovery, mobility, and functional recovery (Chen and Suputtitada).

## Implications

The studies included in this Research Topic collectively support a broader transformation in contemporary pain medicine. Effective pain management increasingly requires integration across rehabilitation medicine, neuroscience, behavioral science, perioperative care, public health, and implementation science. The findings also reinforce the importance of prioritizing functional recovery, participation, independence, and quality of life alongside pain reduction alone.

These evolving approaches align closely with global priorities outlined by the World Health Organization (WHO) Rehabilitation 2030 initiative, the United Nations Decade of Healthy Ageing, and broader efforts toward Universal Health Coverage. Pain management should be viewed not only as symptom control but also as a public health strategy that supports healthy aging, functional independence, workforce participation, social inclusion, and overall societal wellbeing. Integrating evidence-based rehabilitation, prevention, and person-centered care into pain management pathways may contribute substantially to reducing disability, improving population health, and promoting sustainable healthcare systems.

From a healthcare systems perspective, scalable multidisciplinary models, digital rehabilitation platforms, and evidence-based non-pharmacological interventions may help address persistent challenges including healthcare fragmentation, limited specialist access, rising costs, and the growing global burden of chronic musculoskeletal pain. These approaches may be particularly important for aging populations and underserved communities where long-term disability and functional decline substantially affect healthcare utilization and societal participation.

## Future directions

Future pain management will likely move toward increasingly accessible, affordable, personalized, multimodal, and neuroscience-informed models of care integrating rehabilitation, behavioral science, regenerative medicine, digital health, and implementation science. Advances in pain neurobiology, central sensitization research, precision rehabilitation, and translational neuroscience may further improve the ability to tailor interventions according to individual functional, psychological, and biological profiles.

At the same time, future research should continue to prioritize methodological rigor, long-term functional outcomes, accessibility, cost-effectiveness, implementation strategies, and health equity. The continued growth of high-quality systematic reviews and meta-analyses reflects the increasing maturity of evidence-based pain research and will continue to play an important role in strengthening the evidence base, informing clinical guidelines, and identifying future research priorities in pain management (Zou et al.). While randomized controlled trials remain important where feasible, many procedural, rehabilitation, and complex multimodal interventions may also require complementary real-world research methodologies. Large retrospective and prospective cohort studies, pragmatic clinical trials, registry-based studies, adaptive trial designs, mixed-methods research, and implementation science frameworks may better capture clinical complexity, patient preferences, operator-dependent factors, long-term safety, and functional recovery in real-world healthcare systems. In particular, large-scale observational cohorts involving thousands of patients may provide valuable translational and practice-based evidence for interventions that are difficult to standardize or blind within conventional randomized trial designs.

Future research should also focus on optimizing diagnostic pathways by promoting the appropriate use of diagnostic imaging alongside comprehensive clinical assessment and functional evaluation, ensuring that structural findings are interpreted within the broader biopsychosocial context to support patient-centered, function-oriented care (Chen and Suputtitada; Brindisino et al.).

Ultimately, the future of pain management lies not only in reducing pain intensity, but in restoring movement, function, participation, independence, and meaningful living across diverse populations and healthcare systems.

